# The relationship between acromegaly and hepatic steatosis: insights from FibroScan imaging

**DOI:** 10.1007/s40618-025-02799-8

**Published:** 2026-02-20

**Authors:** Tugce Apaydin, Haluk Tarik Kani, Caglayan Keklikkiran, Yusuf Yilmaz, Dilek Gogas Yavuz

**Affiliations:** 1 Department of Endocrinology and Metabolism, Marmara University School of Medicine, Istanbul, Turkey; 2 Department of Gastroenterology, Marmara University School of Medicine, Istanbul, Turkiye, Turkey; 3 Department of Gastroenterology, Recep Tayyip Erdogan University School of Medicine, Rize, Turkey

**Keywords:** Acromegaly, Growth hormone, Hepatic fibrosis, MASLD

## Abstract

**Purpose:**

The relationship between metabolic dysfunction-associated steatotic liver disease (MASLD) and acromegaly is unclear due to the complex metabolic effects of growth hormone (GH). GH stimulates gluconeogenesis, glycogenolysis, and lipolysis, increasing free fatty acids, a risk factor for MASLD, but may also protect against hepatic fat accumulation. In this study, we aimed to evaluate the impact of acromegaly and GH levels on liver steatosis and fibrosis using non-invasive FibroScan imaging.

**Methods:**

This cross-sectional study included 58 patients with acromegaly, 61 age- and sex-matched metabolically comparable controls, and 82 healthy controls. Hepatic steatosis and fibrosis were assessed via controlled attenuation parameter (CAP) and liver stiffness measurement (LSM) by vibration-controlled transient elastography. Moderate to severe steatosis was defined as CAP > 260 dB/m, and significant fibrosis as LSM ≥ 8.0 kPa.

**Results:**

Both acromegaly patients and metabolically healthy individuals exhibited lower CAP (241.8 ± 50.0 and 231.7 ± 51.9 dB/m) and LSM (4.7 ± 1.4 and 4.8 ± 1.8 kPa) scores than metabolically matched controls (CAP 281.7 ± 61.2 dB/m; LSM 5.5 ± 1.8 kPa; CAP *p* < 0.001; LSM *p* = 0.012). Moderate-to-severe steatosis was observed in 36.2% of acromegaly patients, 27.7% of healthy individuals, and 68.8% of metabolically matched controls (*p* < 0.001), while significant liver fibrosis occurred in 5.2%, 7.3%, and 14.7%, respectively (*p* = 0.154). GH levels were lower in acromegaly patients with MASLD (*p* < 0.001) compared to those without. CAP correlated negatively with GH and positively with BMI, waist circumference, and triglycerides, whereas LSM correlated positively with age, BMI, and triglycerides.

**Conclusions:**

Hepatic steatosis was less frequent in acromegaly than in metabolically comparable individuals, and GH was inversely associated with hepatic steatosis scores, possibly suggesting a protective role of GH independent of metabolic comorbidities.

## Introduction

Acromegaly is characterized by excessive secretion of growth hormone (GH), due to a GH-secreting pituitary adenoma, and is frequently associated with comorbid diseases such as hypertension, glucose intolerance and diabetes mellitus [1]. GH hypersecretion induces insulin resistance, which contributes to impaired glucose tolerance, and diabetes mellitus. This occurs through several mechanisms, including reduced peripheral glucose uptake, increased hepatic gluconeogenesis, and enhanced lipolysis and lipid turnover, resulting in elevated circulating free fatty acid levels [2, 3]. Elevated plasma free fatty acids further exacerbate GH-induced insulin resistance [4]. These metabolic alterations lead to changes in fat distribution, characterized by a reduction in total body fat and visceral adiposity, alongside increased ectopic fat deposition in muscle [5, 6]. This distinctive pattern, referred to as *acromegaly-specific lipodystrophy*, represents a unique alteration in body composition among patients with acromegaly [6].

Beyond its metabolic effects, GH also plays a crucial role in regulating liver function and lipid metabolism. GH directly inhibits lipogenesis in hepatocytes, while insulin-like growth factor 1 (IGF-1) promotes cellular senescence in hepatocytes, leading to the inactivation of hepatic stellate cells and the subsequent attenuation of liver fibrosis [7, 8].

Metabolic dysfunction-associated steatotic liver disease (MASLD), formerly known as non-alcoholic fatty liver disease (NAFLD), is the hepatic manifestation of metabolic syndrome, characterized by excessive lipid accumulation in the liver [9]. MASLD can progress to fibrosis, cirrhosis, and hepatocellular carcinoma. MASLD is primarily associated with obesity, insulin resistance, diabetes mellitus, and dyslipidemia, as well as various endocrinopathies [9, 10].

Although acromegaly is frequently associated with insulin resistance, diabetes, and dyslipidemia, studies have shown that patients with active acromegaly tend to have lower intrahepatic lipid levels, likely due to GH-driven stimulation of hepatic ATP synthesis [11]. Moreover, after acromegaly is controlled, hepatic adiposity appears to increase [12, 13]. In contrast, impaired GH axis activity, including GH deficiency or GH resistance syndromes such as Laron syndrome, has been linked to increased intrahepatic lipid accumulation and a higher prevalence of MASLD [8, 14].

Liver biopsy remains the gold standard for diagnosing MASLD; however, due to its invasive nature and associated risks, non-invasive imaging modalities have become increasingly practical for assessing liver fat content and stiffness, such as liver stiffness measurement (LSM) using FibroScan (vibration-controlled transient elastography), magnetic resonance elastography (MRE) [15, 16]. FibroScan has been widely used to measure liver stiffness and steatosis in MASLD and chronic viral hepatitis patients. Studies have reported low measurement error rates and shown to be more sensitive method for detecting lower amounts of hepatic steatosis compared to ultrasonography [17].

The link between MASLD and acromegaly remains uncertain due to GH’s complex metabolic effects. GH stimulates gluconeogenesis, glycogenolysis, and lipolysis, leading to increased free fatty acids, a known MASLD risk factor, on the other hand its lipolytic action may also have protective effects. Based on this hypothesis, we aimed to assess the impact of acromegaly and GH on liver steatosis and fibrosis using FibroScan imaging.

## Methods

### Selection of patients

Fifty-eight patients with acromegaly followed up at Marmara University Education & Research Hospital outpatient clinic who were volunteered for the study, were enrolled cross-sectionally. Among the 58 patients, 13 had active acromegaly, 15 were in remission after the transsphenoidal surgery (TSS), and the remaining 30 patients had age-sex-normalized normal serum IGF-1 levels under somatostatin receptor ligand (SRLs) therapy. A total of 57 patients underwent TSS, while one patient who declined surgery, underwent gamma-knife surgery as a primary treatment.

Sixty-one age-sex matched individuals who were followed in the outpatient clinic for thyroid nodule (euthyroid), nonfunctional adrenal adenoma, prediabetes, T_2_DM and weight gain were included as the control group. An additional 82 metabolically healthy individuals without diabetes, hyperlipidemia, or other metabolic diseases were included as a healthy control group.

Subjects with any secondary cause for hepatic fat accumulation were excluded from both groups, including those with significant alcohol consumption (> 30 g/day for men and > 20 g/day for women), chronic viral hepatitis, autoimmune liver disease, drug-induced steatosis (e.g., use of systemic steroids, tamoxifen and methotrexate), Wilson’s disease, hemochromatosis, and subjects with any malignancy, chronic renal failure, chronic respiratory failure, cardiac disease, GH deficiency, hypogonadism, hypothyroidism and adrenal insufficiency. Additionally, diabetic patients receiving medications known to affect hepatic steatosis (e.g., GLP-1 receptor agonists or pioglitazone) were excluded.

The study was approved by the Local Ethics Committee (09.2020.1346) and conducted in accordance with the Declaration of Helsinki and International Conference on Harmonization Good Clinical Practice guidelines. Written informed consent was obtained from all participants.

### Evaluation of disease activity

The diagnosis of acromegaly was made according to the Endocrine Society guidelines [18], based on clinical features, the presence of a pituitary adenoma on magnetic resonance imaging (MRI), and elevated serum IGF-1 levels exceeding 1.3 times the upper normal limit. Hormonal remission was considered when subjects had age-normalized serum IGF-1 value and suppression of serum GH levels (< 0.4 µg/L) during the oral glucose tolerance test (OGTT). Both remissions after TSS and biochemically controlled patients with SRLs were considered as controlled acromegaly (CA). Active acromegaly (AA) is accepted when IGF-1 levels are not normalized for age despite the medical, surgical, and/or radiation treatment [19]. Median duration of the active disease after diagnosis was 87 months (min–max: 3–216) in AA, and 122 months (min–max:10–360) in CA (*p* = 0.110). CA patients had been in remission for a median of 36 months (range: 9–86 months) at the time of inclusion.

### Clinical and laboratory evaluation

Demographic data (age and sex), medical histories, medications, and laboratory data including fasting plasma glucose (FPG), glycated hemoglobin (HbA1c), insulin levels, low-density lipoprotein cholesterol (LDL-c), high-density lipoprotein cholesterol (HDL-c), triglyceride, GH, and IGF-1 were obtained from medical records. Blood samples were collected in the morning following a minimum of 8 h of fasting.

Height and weight were measured, and the body mass index (BMI) was calculated as weight (kg) divided by height squared (m^2^). Waist circumference (WC) was measured in the standing position in the middle of the iliac crest and the lower costal margin.

FPG levels were measured using an enzymatic UV test (hexokinase method) on a Beckman Coulter analyzer (reference range: 65–100 mg/dL). HbA1c was analyzed by boronate affinity high-performance liquid chromatography (Premier Hb9210, Trinity Biotech, USA; reference range: 4.5%–5.7%). GH (reference range: 0–2.5 µg/L) and IGF-1 were measured using a chemiluminescent immunometric assay (Immulite 2000, Siemens, USA). GH levels were measured at three different time points via an intravenous catheter with delayed collection, and the mean of these measurements was used for subsequent analysis. Total cholesterol (reference range: 80–200 mg/dL), HDL-c (35–70 mg/dL), and triglycerides (30–100 µg/L) were measured using an enzymatic colorimetric method on an AU5800 analyzer (Beckman Coulter, USA).

### Noninvasive hepatosteatosis scores

The Hepatic Steatosis Index (HSI), Fatty Liver Index (FLI), and Triglyceride-Glucose (TyG) index were used as non-invasive markers to evaluate hepatic steatosis, while the Fibrosis-4 (FIB-4) index was used to assess liver fibrosis.HSI was calculated as: HSI = 8 × (ALT/AST) + BMI + 2 (if female) + 2 (if diabetic). FLI was calculated as FLI = e^*x*^/(1 + e^*x*^) × 100,where *x* = 0.953 × log_e_(TG) + 0.139 ×BMI + 0.718 × log_e_(GGT) + 0.053 × (WC) − 15.745. TyG index was calculated as: Ln(fasting triglycerides [mg/dL] x fasting glucose [mg/dL])/2. The FIB-4 index was calculated using the following formula: (Age [years] × AST [U/L])/(Platelet count [10⁹/L] × √ALT [U/L].

### Assessment of liver stiffness measurement by transient elastography

Hepatic steatosis and fibrosis were assessed using controlled attenuation parameter (CAP) and liver stiffness measurement (LSM) via FibroScan 502 Touch device (Echosens SA, Paris, France). A single operator performed exams following at least three hours of fasting, in accordance with the manufacturer’s guidelines. During the examinations, patients were positioned in the dorsal decubitus position, and the M-probe (3.5 Hz) was initially used, with automatic transition to the XL probe (2.5 Hz) when required. Reliable transient elastography (TE) was defined as ≥ 10 valid measurements with an interquartile range-to-median ratio ≤ 0.3 [20]. Steatosis severity was categorized as mild (11%–33% fat, 238–259 dB/m), moderate (34%–66% fat, 260–292 dB/m), or severe (> 67% fat, ≥ 293 dB/m) [21, 22]. Significant fibrosis (F ≥ 2) was determined based on a liver stiffness measurement (LSM) cutoff of ≥ 8 kPa [9].

### Statistical analysis

Data were tested for normality using appropriate distribution tests. For normally distributed continuous variables, comparisons between groups were performed using the student’s t-test, while the Mann–Whitney U test was applied for non-normally distributed data. For comparisons among three groups, one-way ANOVA or the Kruskal–Wallis test was used as appropriate, followed by post hoc pairwise comparisons. Continuous data presented as mean ± standard deviation (SD). Categorical variables were summarized using counts and percentages. Categorical data were analyzed using the Chi-square (χ^2^) test or Fisher’s exact test as appropriate. The correlation between numerical variables was tested with Spearman’s correlation test. Univariate logistic regression analyses and multiple linear regression analyses were performed to identify independent predictors of CAP and LSM values. All variables were tested for multicollinearity prior to inclusion, and variables with clinical relevance and statistical significance in univariate analyses were selected for the final models. The results were evaluated at 95% confidence intervals (CI), and *p* < 0.05 was considered statistically significant. All statistical analyses were performed using statistical software (GraphPad InStat 3.0; GraphPad Software, Inc., San Diego, CA, USA).

## Results

### Patients’ distributions, characteristics and biochemical parameters

Clinical and laboratory characteristics of the acromegaly and metabolically matched control groups are summarized in Table 1. The two groups showed similar distributions in terms of age, sex, WC, BMI, and the prevalence of diabetes and hypertension (Table 1). There were no significant differences in fasting blood glucose or HbA1c levels (*p* > 0.05). However, the control group had significantly higher levels of total cholesterol (*p* = 0.007), triglycerides (*p* = 0.007), ALT (*p* = 0.008), and AST (*p* = 0.004).

**Table 1:** Comparison of general characteristics between patients with acromegaly and metabolically matched controls

	Acromegaly *n* = 58	Metabolically Matched controls *n* = 61	*p*
Age, yrs	49.9 ± 11.7	46.2 ± 10.5	0.073
Female sex, n (%)	32 (55.1)	30 (49.2)	0.990
T2DM, n (%)	25 (44.1)	20 (32.8)	0.262
HT, n (%)	22 (37.9)	17 (27.8)	0.328
HL, n (%)	20 (34.5)	20 (32.7)	0.849
Smoking, n (%)	8 (13.8)	15 (24.5)	0.166
Waist circumference, cm	100.8 ± 12.8	103.7 ± 16.7	0.316
BMI, kg/m^2^	30.4 ± 5.4	32.2 ± 6.5	0.107
FPG, mg/dL	106.2 ± 29.4	115.3 ± 48.2	0.230
HbA1c, %	6.1 ± 0.9	6.4 ± 1.7	0.142
LDL-C, mg/dL	106.0 ± 32.5	117.6 ± 34.2	0.065
Total cholesterol, mg/dL	177.6 ± 37.0	196.6 ± 37.8	**0.007**
Triglyceride, mg/dL	112.4 ± 63.4	147.1 ± 73.2	**0.007**
HDL-C, mg/dL	50.2 ± 13.5	47.8 ± 15.0	0.388
ALT, U/L	17.0 ± 9.1	22.3 ± 11.1	**0.008**
AST, U/L	17.2 ± 6.2	22.6 ± 12.3	**0.004**
GGT, U/L	21.8 ± 18.1	27.9 ± 18.4	0.089
INR	1.1 ± 0.1	1.0 ± 0.1	0.285
Platelets, ×10^3^/µl	238.5 ± 60.9	261.1 ± 73.1	0.076
CAP, dB/m	241.8 ± 50.0	281.7 ± 61.2	**< 0.001**
LSM, kPa	4.7 ± 1.4	5.5 ± 1.8	**0.008**
Moderate-severe hepatic steatosis, n (%)	21 (36.2)	42 (68.8)	**< 0.001**
Significant liver fibrosis, n (%)	3 (5.1)	9 (14.7)	0.152

Values were reported as mean ± SD

BMI, Body mass index; CAP, controlled attenuation parameter; T2DM, type 2 diabetes mellitus; FPG, fasting plasma glucose; HDL-C, high-density cholesterol; HL, hyperlipidemia; HT, hypertension; LDL-C, low-density cholesterol; LSM, liver stiffness measurement; ULN, upper limit of normal

Hepatic steatosis index and fatty liver index did not differ significantly between groups (40.5 ± 6.9 vs. 42.8 ± 7.8, *p* = 0.130; 57.0 ± 32.5 vs. 69.2 ± 32.6, *p* = 0.065, respectively). FIB-4 scores were also similar (1.0 ± 0.5 vs. 0.9 ± 0.3, *p* = 0.142). In contrast, triglyceride-glucose index (TyG) was significantly higher in the metabolically matched control group (4.6 ± 0.2 vs. 4.8 ± 0.3, *p* < 0.001).

### Assessment of liver steatosis and fibrosis via fibroscan

The CAP score, a marker of hepatic steatosis, was significantly lower in the acromegaly group than in controls (241.8 ± 50.0 dB/m vs. 281.7 ± 61.2 dB/m, *p* < 0.001). Similarly, the LSM score, used to assess hepatic fibrosis, was lower in the acromegaly group (4.7 ± 1.4 kPa vs. 5.5 ± 1.8 kPa, *p* = 0.008). Mild steatosis was detected in 8 (13.8%) patients, moderate steatosis in 14 (24.1%) patients, and severe steatosis in 7 (12.1%) patients. Moderate to severe hepatic steatosis was present in 36.2% (*n* = 21) of the acromegaly patients and 68.8% (*n* = 42) of the control group (*p* = 0.005).

To evaluate whether these findings were also present in the metabolically healthy group, an additional control group of 82 individuals (mean age 37.8 ± 11.1 years; 59.8% women) was included. CAP and LSM scores differed significantly among the three groups (CAP *p* < 0.001; LSM *p* = 0.021). Post hoc pairwise comparisons showed that CAP and LSM were lower in the metabolically healthy group than in the metabolically matched control group (CAP: 231.7 ± 51.9 dB/m vs. 281.7 ± 61.2 dB/m, *p* < 0.001; LSM: 4.8 ± 1.8 kPa vs. 5.5 ± 1.8 kPa, *p* < 0.05), but similar to those of patients with acromegaly (CAP: 231.7 ± 51.9 dB/m vs. 241.8 ± 50.0 dB/m; LSM: 4.8 ± 1.8 kPa vs. 4.7 ± 1.4 kPa, *p* = 0.120). Moderate-to-severe hepatic steatosis was present in 27.7% (*n* = 23) of the metabolically healthy group. Hepatic steatosis prevalence differed among the three groups (*p* < 0.001), being higher in the metabolically matched control group than in both the acromegaly and metabolically healthy groups (*p* < 0.001), with no significant difference between the acromegaly and metabolically healthy groups (*p* = 0.204). Additionally, no significant difference was observed in the prevalence of hepatic fibrosis among the three groups (*p* = 0.154), with fibrosis present in 5.1% (*n* = 3) of acromegaly patients, 14.7% (*n* = 9) of metabolically matched controls, and 7.3% (*n* = 6) of healthy individuals (Table 2).

**Table 2:** Comparison of the fibroscan measurements among the three groups

	Acromegaly *n* = 58	Metabolically Matched Controls *n* = 61	Healthy Controls *n* = 82	*p*
Age, yrs	49.9 ± 11.7	46.2 ± 10.5	37.8 ± 11.1	**< 0.001**
Female sex, n (%)	32 (55.1)	30 (49.2)	49 (59.8)	0.453
BMI, kg/m^2^	30.4 ± 5.4	32.2 ± 6.5	26.6 ± 5.1	**< 0.001**
CAP	241.8 ± 50.0	281.7 ± 61.2	231.7 ± 51.9	**< 0.001**
LSM, kPa	4.7 ± 1.4	5.5 ± 1.8	4.8 ± 1.8	**0.012**
Moderate-severe hepatic steatosis, n (%)	21 (36.2)	42 (68.8)	23 (27.7)	**< 0.001**
Significant liver fibrosis, n (%)	3 (5.1)	9 (14.7)	6 (7.3)	0.154

Values were reported as mean ± SD

BMI, Body mass index; CAP, controlled attenuation parameter; LSM, liver stiffness measurement

GH levels were significantly lower in acromegaly patients with MASLD compared to those without MASLD (2.1 ± 1.8 µg/L vs. 0.70 ± 0.6 µg/L, *p* < 0.001), whereas no difference was observed in IGF-I levels (203.6 ± 133.3 ng/mL vs. 166.4 ± 65.5 ng/mL, *p* = 0.183).

### Evaluation of acromegaly patients according to the disease status

Of the 58 patients, 13 (22.4%) were uncontrolled, 45 (77.6%) were controlled (of whom 15 were in remission after TSS and 30 were biochemically controlled). Mean GH, IGF-1 and IGF-1 ULN ratio were 1.5 µg/L (min-max: 0.05–10.9), 188.5 ng/mL (min-max:57–686), 0.86 (min-max:0.13–2.32), respectively.

There were no significant differences between patients with AA and CA groups in terms of BMI, WC, FPG, HbA1c, LDL-c and triglyceride levels (Table 3). As expected, GH and IGF-1 levels were significantly lower in CA group compared to AA group [1.04 ± 0.9 µg/L (min-max: 0.04–3.3) vs. 3.4 ± 3.0 µg/L (min-max: 1–10.6); *p* < 0.001; and 145.4 ng/mL (min-max: 57–226) vs. 325.1 ng/mL (min–max: 206–686); *p* < 0.001]. Similarly, the IGF-1 ULN ratio was lower in CA patients [0.7 ± 0.2 (0.1–1.0) vs. 1.4 ± 0.3 (1.1–2.3), *p* < 0.001]. CAP and LSM scores did not significantly differ between the groups (*p* > 0.05).

**Table 3:** Comparison of patients with active acromegaly and controlled acromegaly

	Active Acromegaly *n* = 13	Controlled Acromegaly *n* = 45	*p*
Age, yrs	44.7 ± 14.0	51.4 ± 10.7	0.071
Waist circumference, cm	102.7 ± 12.7	95.8 ± 12.0	0.144
BMI, kg/m^2^	30.5 ± 5.2	30.1 ± 6.1	0.825
IGF-1, ng/mL	325.1 ± 139.1	145.4 ± 42.4	**< 0.001**
IGF-1 ULN, %	1.4 ± 0.3	0.7 ± 0.2	**< 0.001**
Basal GH, µg/L	3.4 ± 3.0	1.04 ± 0.9	**< 0.001**
FPG, mg/dL	108.1 ± 38.6	99.5 ± 19.0	0.357
HbA1c, %	6.1 ± 0.9	5.5 ± 0.5	0.165
LDL-C, mg/dL	106.6 ± 33.5	104.0 ± 30.4	0.808
Total cholesterol, mg/dL	179.1 ± 37.8	173.6 ± 35.3	0.639
Triglyceride, mg/dL	95.7 ± 33.9	117.2 ± 69.1	0.286
HDL-C, mg/dL	50.3 ± 14.2	50.0 ± 11.0	0.939
ALT, U/L	16.8 ± 9.8	17.6 ± 6.4	0.804
AST, U/L	17.4 ± 6.8	16.6 ± 3.0	0.681
GGT, U/L	18.2 ± 7.9	20.8 ± 13.6	0.551
INR	1.0 ± 0.1	1.0 ± 0.1	0.938
Platelets, ×10^3^/µl	238.0 ± 52.0	238.7 ± 63.8	0.970
CAP, dB/m	234.7 ± 56.6	243.8 ± 48.4	0.564
LSM, kPa	4.7 ± 0.9	4.7 ± 1.5	0.963

Values were reported as mean ± SD

BMI, Body mass index; CAP, controlled attenuation parameter; T2DM, type 2 diabetes mellitus; FPG, fasting plasma glucose; GH, growth hormone; HDL-C, high-density cholesterol; IGF-1, insulin-like growth factor 1; HL, hyperlipidemia; HT, hypertension; LDL-C, low-density cholesterol; LSM, liver stiffness measurement; ULN, upper limit of normal

No significant differences were observed in CAP (248.3 ± 38.2 dB/m vs. 241.6 ± 53.3 dB/m, *p* = 0.668) score and LSM (5.06 ± 1.6 kPa vs. 4.6 ± 1.55 kPa, *p* = 0.363) values between the group receiving SRLs and the group in remission after surgery.

### Liver steatosis and fibrosis scores according to the presence of diabetes and dyslipidemia

CAP and LSM values were significantly higher in patients with diabetes compared to those without diabetes in both the acromegaly group (257.2 ± 44.0 dB/m vs. 230.1 ± 51.7 dB/m, *p* = 0.039; 5.3 ± 1.3 kPa vs.4.3 ± 1.3 kPa, *p* = 0.005) and the control group (318.4 ± 46.6 dB/m vs.263.3 ± 59.7 dB/m, *p* < 0.001; 6.3 ± 1.9 kPa vs.5.1 ± 1.5 kPa, *p* = 0.012). After excluding individuals with diabetes, there were a significant difference in CAP score and LSM values remained significantly lower in the acromegaly (*n* = 33) compared to control (*n* = 41) group (230.1 ± 51.7 dB/m vs. 263.3 ± 59.7 dB/m, *p* = 0.014; 4.3 ± 1.3 kPa vs. 5.1 ± 1.5 kPa, *p* = 0.017). Similarly, among individuals with diabetes, CAP scores were significantly lower in patients with acromegaly than in diabetic controls (257.2 ± 44.0 vs. 318.4 ± 46.6 dB/m, *p* < 0.001), whereas no statistically significant difference was observed in LSM values (5.3 ± 1.3 vs. 6.4 ± 2.0 kPa, *p* = 0.060).

Among patients with acromegaly, there was no significant difference in CAP or LSM values between those with and without hyperlipidemia (250.9 ± 44.1 dB/m vs. 237.0 ± 52.7 dB/m, *p* = 0.318; 5.2 ± 1.5 kPa vs. 4.4 ± 1.3 kPa, *p* = 0.084). However, in the control group, individuals with hyperlipidemia had significantly higher CAP and LSM values (318.6 ± 44.8 dB/m vs. 263.2 ± 60.3 dB/m, *p* < 0.001; 6.3 ± 1.8 kPa vs.5.1 ± 2.8 kPa, *p* = 0.024). Even after excluding individuals with hyperlipidemia, acromegaly patients (*n* = 38) continued to show lower CAP and LSM values compared to controls (*n* = 41) (233.8 ± 49.6 dB/m vs. 263.2 ± 60.3 dB/m, *p* = 0.022; 4.4 ± 1.3 kPa vs. 5.2 ± 1.7 kPa, *p* = 0.040).

### Correlation analyses and multiple regression analyses

In acromegaly patients, a negative correlation was found between CAP score and basal GH levels (r:−0.311, *p* = 0.017), while positive correlations were identified between CAP score and BMI (r:0.409, p: 0.001), WC (r:0.367, p:0.007), FPG (r: 0.302, p:0.020), triglyceride (r:0.355, p: 0.005), ALT (r:0.321, p:0.013), hepatic steatosis index (r:0.505, *p* < 0.001), fatty liver index (r:0.367, *p* = 0.014) and TyG (r:0.418, *p* = 0.001).

LSM values was positively correlated with age (r:0.399, p:0.001), BMI (r:0.500, *p* < 0.001), triglyceride (r:0.266, p:0.041), ALT (r:0.397, p:0.001), AST (r: 0.298, p: 0.021), hepatic steatosis index (r:0.491, *p* < 0.001), and TyG (r:0.413, *p* = 0.001).

Univariate regression analyses showed that the presence of T_2_DM or hyperlipidemia was not associated with CAP and LSM values (*p* > 0.05).

In multiple regression analyses, growth hormone (GH) level was identified as an independent predictor of CAP scores (R² = 40.1%, *p* < 0.001). Additionally, age, body mass index (BMI), and GH levels were independent predictors of LSM values (R² = 52.4%, *p* < 0.001) (Table 4).

**Table 4:** Multiple regression analyses

Variable	Dependent variable	Independent variable	*R* ^2^	*p*
Model 1	CAP Score	Age, BMI, Waist circumference, FPG, HbA1c, triglyceride, ALT, GH **(*p* = 0.023)**	40.1%	< 0.001
Model 2	LSM value	Age **(*p* < 0.001)**, BMI **(*p* = 0.004)**, Waist circumference, FPG, HbA1c, triglyceride, ALT, AST, GH **(*p* = 0.004)**	52.4%	< 0.001

BMI, Body mass index; CAP, controlled attenuation parameter; FPG, fasting plasma glucose; GH, growth hormone; LSM, liver stiffness measurement

## Discussion

In this study, hepatic steatosis was less frequent in patients with acromegaly than in the metabolically matched control group, and similar to healthy control group. Both, CAP and LSM scores were significantly lower in the acromegaly group compared to metabolically matched controls, regardless of diabetes mellitus or hyperlipidemia, and were comparable to the healthy control group. No significant differences were observed between the groups in commonly used non-invasive MASLD assessment tools, including the Fatty Liver Index, Hepatic Steatosis Index, and FIB-4 score, with the exception of the TyG. While a negative correlation was observed between GH levels and hepatic steatosis. BMI, WC, FPG, and triglyceride levels were positively correlated. On the other hand, liver fibrosis was associated with age, BMI, and GH levels.

In our study, 50% of acromegaly patients and 46.9% of healthy controls exhibited MASLD, with 36.2% and 27.7% showing moderate to severe hepatic steatosis,- a relatively high rate compared to the 38% prevalence reported in the general population [23]. In the studies by Koutsou-Tassopoulou et al. and Ciresi et al., MASLD was detected in 66% and 62% of acromegaly patients, respectively, based on ultrasonographic assessment [24, 25]. Eroglu et al. found a lower prevalence of MASLD (32%) using magnetic resonance imaging [26]. Coskun et al. reported a 26.1% prevalence using shear wave elastography [27]. The differences between studies may be explained by differences in the diagnostic methods and examiners across studies.

Bredella et al. and Winhofer et al., who studied AA subjects using magnetic resonance spectroscopy, demonstrated that individuals with acromegaly had lower intrahepatic lipid (IHL) compared to controls, despite decreased insulin sensitivity and hyperinsulinemia [2, 5, 11]. Similarly, in our study, both AA and CA patients showed lower CAP scores than control group. Moreover, Bredella et al. reported a significant increase in IHL following biochemical control of acromegaly [5], and Reyes-Vidal et al. also observed a rise in IHL one year after surgery [12, 25]. Conversely, Kuker et al. found that IHL remained low in the long term after surgical intervention [28]. In Eroglu et al.’s study magnetic resonance imaging-proton density fat fraction measurements showed lower IHL levels in AA compared to CA patients [26]. In contrast, in our study, CAP and LSM values did not significantly differ between AA and CA subjects. This discrepancy between studies may be attributed to differences in the duration of remission among patients included in each study. In our cohort, remission duration ranged from 9 to 86 months, and in some patients, the relatively short period following biochemical control may not have been sufficient to allow for significant changes in visceral adiposity. While GH excess reduces fat via lipolysis, its normalization increases visceral fat, potentially offsetting improvements in hepatic steatosis and fibrosis markers. Thus, the lack of difference in hepatic parameters may also reflect complex metabolic changes following disease control.

Limited research is available on liver fibrosis and the findings are often contradictory. While Eroglu et al. reported similar liver stiffness across groups [26], our study observed lower LSM values in acromegaly patients, although the frequency of liver fibrosis did not reach statistical significance. Conversely, Coskun et al. reported an increased prevalence of liver fibrosis in newly diagnosed acromegaly patients [27].

We observed a negative correlation between hepatic steatosis and GH levels. Similarly, GH levels have been inversely correlated with hepatic lipid accumulation in obese women and healthy controls [29]. Previous studies have shown that individuals with GH deficiency, including those with Laron syndrome, are more prone to developing hepatic steatosis, and chronic IGF-I replacement has not been shown to improve MASLD [30]. Furthermore, GH replacement therapy in subjects with GH deficiency resulted in significant improvement in NASH, histological changes, serum liver enzyme levels, fibrotic markers, and lipid abnormalities, accompanied by a marked reduction in oxidative stress, suggesting a direct action of GH in preventing steatosis in hepatocytes [14, 31].

Patients with MASLD have been shown to exhibit lower baseline and stimulated GH levels in non-acromegalic populations [10], and similarly, in our study, patients with MASLD also demonstrated lower GH levels. Recombinant GH therapy has been also reported to improve fatty liver disease in individuals with overweight and obesity [32–34]. Additionally, tesamorelin, a GH-releasing hormone analog, has been shown to significantly reduce IHL levels and slow fibrosis progression in HIV-positive patients with NAFLD [35]. In contrast, treatment with GH receptor antagonist pegvisomant in acromegaly patients have been found to be associated with increased hepatic steatosis [36]. Notably, none of the subjects in our study were receiving pegvisomant therapy. In our study, the use of SRLs did not appear to have a distinct impact on hepatic steatosis or fibrosis scores compared to the group in remission following TSS.

MASLD has become as the most common cause of chronic liver disease and is frequently associated with risk factors like obesity, metabolic syndrome, type 2 diabetes, and hyperlipidemia [9]. In our study, the prevalence of diabetes mellitus and hyperlipidemia was comparable between the control group and patients with acromegaly. Steatosis and fibrosis were found to be less common in the acromegaly group, independent of diabetes and hyperlipidemia status. BMI, a widely used measure of adiposity, remains one of the strongest risk factors for MASLD [37]. Consistent with findings in the general population, hepatic steatosis in our study was positively associated with BMI, waist circumference, and triglyceride levels. Similarly, Coskun et al. reported significant correlations between these parameters and hepatic steatosis [27], whereas Eroglu et al. observed a positive correlation only with triglyceride levels [26]. One reason behind our findings may be that the lower CAP and LSM scores observed in the acromegaly group were related to their lower triglyceride levels compared to the control group.

Several noninvasive predictive scores—such as the fatty liver index, hepatic steatosis index, TyG, and visceral adiposity index—are frequently used to assist in the evaluation of MASLD; while scoring systems like APRI and FIB-4 are commonly employed to estimate fibrosis risk. In our study, the TyG was significantly higher in the control group, whereas the other scores were similar between the groups. In the study by Eroglu et al., the visceral adiposity index, fatty liver index, and TyG showed adequate diagnostic performance in patients with acromegaly [26]. Hepatic steatosis index was shown to decrease after 12 months of treatment with SRLs [25].

This study has several limitations. First, the small AA sample, due to the rarity of acromegaly, limits definitive conclusions; larger patient cohorts and long-term prospective studies are needed to assess the impact of disease activity on hepatic fibrosis. Second, since the BMI of individuals in both groups was above 30 kg/m², an XL probe—commonly used in obese individuals to reduce diagnostic inaccuracies—was used, although it has been reported to be less accurate [38]. Nevertheless, the reliability of FibroScan examinations at our center was 97.25%, with a measurement error rate of only 2.75% [17]. Third, triglyceride levels are known to contribute to the development of MASLD and liver fibrosis [39]. In our metabolically matched control group, higher triglyceride levels may have influenced the elevated steatosis and fibrosis scores observed. However, other metabolic parameters were similarly distributed between the two groups. Fourth, the cross-sectional design precludes establishing a causal relationship regarding the effects of GH. Finally, although we could not completely eliminate the potential influence of antidiabetic medications, the distribution and glycemic control of diabetes were comparable between the control and acromegaly groups.

In conclusion, elevated GH may confer protection against fatty liver in acromegaly patients, with age and BMI playing a greater role in fibrosis risk, independent of diabetes or hyperlipidemia. Factors such as triglyceride levels, BMI, and waist circumference were found to be associated with fatty liver. Liver fibrosis was observed in 5.1% of the acromegaly patients; however, no statistical significance was detected, though this may change with a larger sample size. Larger, long-term studies are needed to further clarify these relationships.

## Abbreviations

ALT: Alanine aminotransferase
ALP: Alkaline phosphatase
AST: Aspartate aminotransferase
BMI: Body mass index
CAP: Controlled attenuation parameter
GGT: Gamma-glutamyl transpeptidase
HDL: High-density lipoprotein
IGF-1: Insulin‐like growth factor 1
LDL: Low-density lipoprotein
LSM: Liver stiffness measurement
MASLD: Metabolic dysfunction-associated steatotic liver disease
WC: Waist circumference
TyG: Triglyceride-glucose index
